# Kawasaki disease shock syndrome complicated by coronary aneurysms: a case report

**DOI:** 10.11604/pamj.2021.38.52.27599

**Published:** 2021-01-18

**Authors:** Ahmed Rassas, Rihab Guizani, Amina Werdani, Nesrine Jammeli, Bahri Mahjoub

**Affiliations:** 1 Department of Pediatrics, Taher Sfar University Hospital, Mahdia, Tunisia,; 2 Department of Ear Nose and Throat (ENT), Taher Sfar University Hospital, Mahdia, Tunisia

**Keywords:** Kawasaki disease, shock, coronary aneurysm, case report

## Abstract

Kawasaki disease is a generalized systemic vasculitis, which primarily affects medium-sized arteries. Kawasaki disease shock syndrome is a rare but severe presentation of this disease. This report describes a case of delayed diagnosis of Kawasaki disease shock syndrome in a 13-year-old boy who presented with cervical adenophlegmon, persistent fever, injected conjunctiva, rash, and hypotension. Echocardiography revealed the presence of bilateral coronary aneurysms. Early recognition of Kawasaki disease shock syndrome can be difficult; however, delay in diagnosis and treatment can increase the risk of coronary artery disease.

## Introduction

Kawasaki disease (KD) is a generalized systemic vasculitis predominantly involving medium-sized arteries [1]. It is the most common cause of acquired heart disease in the pediatric age group and result in permanent damage to coronary arteries in up to 25% of untreated children [2]. The characteristic clinical features of KD are prolonged unexplained fever, accompanied by nonexudative conjunctivitis, rash, inflammation of the lips and oral cavity, cervical lymphadenitis, swollen extremities and periungual desquamation [3]. Kawasaki disease shock syndrome (KDSS) is a rare and severe form of KD. Delay in the diagnosis and treatment of this presentation can lead to more serious cardiac complications. We report here one case of Kawasaki disease shock syndrome (KDSS) complicated by bilateral coronary artery aneurysms.

## Patient and observation

A 12 year-old boy, with no notable medical history, was admitted to Ear Nose and Throat (ENT) department for a 3 days history of fever with painful neck mass. The diagnosis of adenophlegmon was retained and the patient was treated with cefotaxime and metronidazole. After five days of antibiotics, the adenophlegmon regressed but the child kept a high fever. He was then transferred to our pediatric department for exploration.

**Figure 1:**
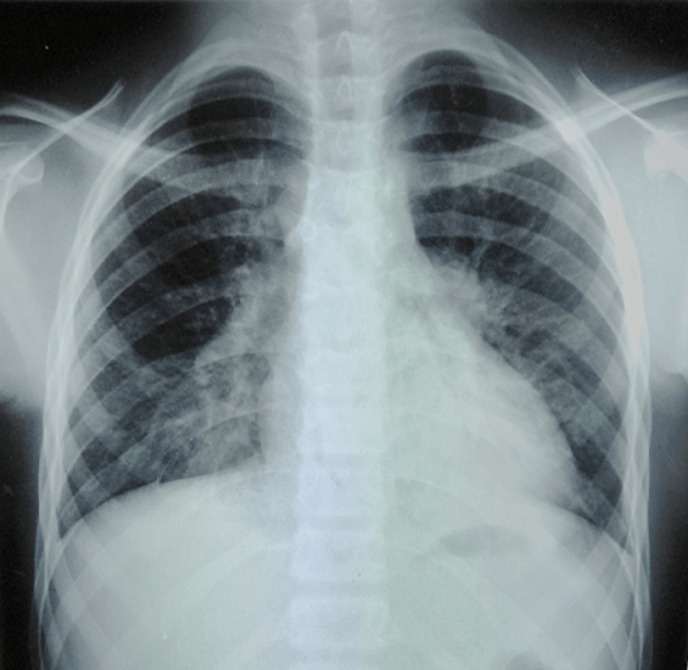
chest X-ray showed normal size heart with bilateral base-predominant alveolar and interstitial infiltrates

**Figure 2:**
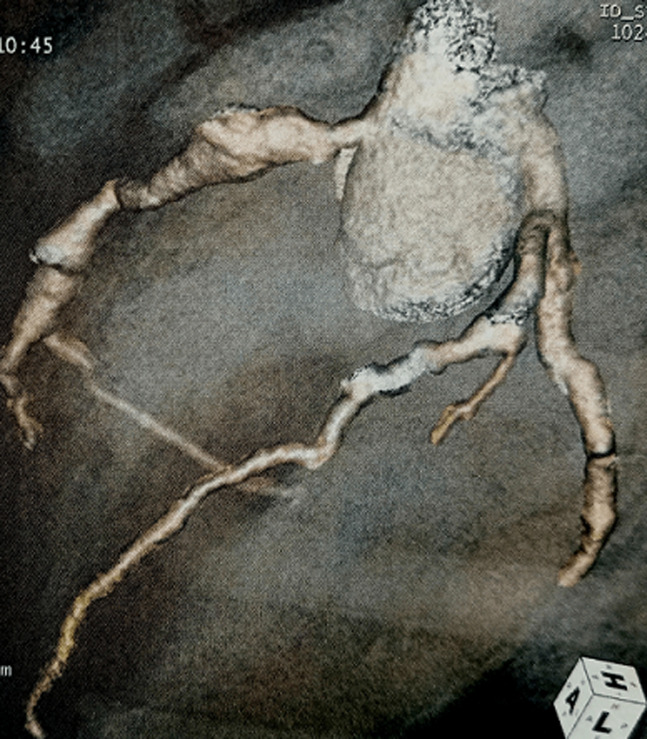
coronary CT angiography revealed multiple coronary aneurysms involving all 3 major coronary arteries

On admission, he was tired and vital signs revealed a temperature of 40^o^C, respiratory rate of 30 breaths per minute, heart rate of 120 beats per minute, blood pressure of 81/40 mmHg, warm extremities, and good capillary refil with normal Oxygen saturation. He had erythematous macules on the trunk and bilateral conjunctival hyperemia. The remainder of the physical examination was unremarkable. The diagnosis of toxic shock syndrome was suspected. The patient received aggressive intravenous fluid hydratation with crystalloids and broad-spectrum antibiotics (vancomycin, cefotaxime and gentamicin) were administered after blood cultures. The chest X-ray showed normal size heart with bilateral base-predominant alveolar and interstitial infiltrates (Figure 1). The initial investigations showed white blood cell (WBC) count of 15100/mm^3^ with 90% neutrophils and platelet (Plt) count of 312000/mm^3^, a C-reactive protein (CRP) of 62 mg/l, and a serum sodium level of 133 mmol/l while other blood tests were within the standard limits. The summary of investigations is shown in Table 1. The patient´s status improved within 12-24 hours. However a low grade fever persisted after 3 days. Multiple blood cultures were negative, the CRP level decreased and the erythrocyte sedimentation rate (ESR) was markedly increased. The echocardiography, performed on the 11^th^ day of illness, showed bilateral coronary artery aneurysms (right coronary artery (RCA): 8 mm and anterior interventricular artery (AIV): 7 mm). The coronary computed tomography (CT) angiography revealed multiple coronary aneurysms involving all 3 major coronary arteries (Figure 2). The diagnosis of KDSS was made a posteriori on the basis of these findings. Intravenous immunoglobulin (2 g/kg) for 24 hours, high-dose acetylsalicylic acid and oral anticoagulation (acenocoumarol) treatment were applied. The fever disappeared after 2 days and the inflammatory markers gradually normalized. The patient was discharged from the hospital with low-dosage (3 mg/kg per day) acetylsalicylic acid and oral anticoagulation. One-year follow up echocardiography showed stable coronary aneurysms without improvement.

**Table 1:** summary of investigations

Day of illness	Day 3	Day 8	Day 11	Day 30
WBC/neutrophils (*103/mm^3^)	14.2/12.8	15.1/13.6	13.5/10.9	7.02/2.7
**Hg (g/dl)**	13.2	12.1	11	12,7
Plt (*103/mm^3^)	246	312	637	274
**CRP (mg/l)**	81	62	15	
**ESR (mm/1^st^H)**			107	13
**Natremia (mmol/l)**	134	133	133	
**AST (IU/L)**	25	40	31	
**ALT (IU/L)**	19	19	46	
**Serum Creatinine (μmol/L)**	82	75		
**Blood urea nitrogen (mmol/l)**	8.1	6.5		

WBC: white blood cell, Hg: hemoglobin, Plt: platelet, CRP: C-reactive protein, ESR: erythrocyte sedimentation rate, AST: aspartate aminotransferases, ALT: alanine aminotransferases

## Discussion

KDSS was first described by Kanegaye *et al*. in 2009. It was defined by the combination of criteria for KD and systolic hypotension (-2DS blood pressure defined for age and sex) or clinical signs of poor perfusion (tachycardia, prolonged capillary refill time, cool extremities, diminished pulse volume, oliguria) [4]. The incidence rate of KDSS varies from 1.23% to 7% [4-7].

The clinical manifestations of KDSS are atypical [8] and its diagnosis is sometimes difficult [1]. In our case, we initially discussed the toxic shock syndrome (TSS) which is an acute exotoxin-mediated multisystem disorder caused by superantigens produced by staphylococcus aureus or streptococcus pyogens infections. It is characterized by fever, rash followed by desquamation, vomiting and diarrhea, hypotension, conjunctivitis and strawberry tongue [9]. The similar presentations of KDSS and TSS may lead to a delayed diagnosis of KD and thus delay in giving intravenous immunoglobulin (IVIG) which leads to a failure in the prevention of coronary artery damage.

The exact cause of severe hypotension in patients with KD is unknown. It is probably multifactorial, possibly including vasculitis with ongoing capillary leakage, myocardial dysfunction, and cytokine dysregulation [1,6]. Inflammatory cytokines are known to damage myocardial cells and work as cardiac depressant [10]. Compared with patients with hemodynamically normal KD, patients with KDSS are older, have more prolonged fever and are more likely to suffer complications such as aseptic meningitis, hepatic failure and renal failure [6]. Patients with KD and shock have also been reported to have higher incidence of coronary artery abnormalities, mitral regurgitation and prolonged myocardial dysfunction [1,4,11]. During KDSS, hypoalbuminemia, hyponatremia, consumption coagulopathy, and electrocardiography (ECG) abnormalities are more common and patients have higher CRP levels, greater proportions of bands, lower hemoglobin levels and lower platelet counts [6,7].

Compared with patients with hemodynamically normal KD, patients with KDSS are older, have more prolonged fever and are more likely to suffer complications such as aseptic meningitis, hepatic failure and renal failure [6]. Patients with KD and shock have also been reported to have higher incidence of coronary artery abnormalities, mitral regurgitation and prolonged myocardial dysfunction [1,4,11]. During KDSS, hypoalbuminemia, hyponatremia, consumption coagulopathy, and ECG abnormalities are more common and patients have higher CRP levels, greater proportions of bands, lower hemoglobin levels and lower platelet counts [6, 7].

The efficacy of IVIG administered in the acute phase of KD in reducing the prevalence of coronary abnormalities is well established. Patients should be treated with IVIG and aspirin. This therapy should be instituted within the first 10 days of illness and, if possible, within 7 days of illness. However, children who present often 10 days of fever still should be treated if fever or other signs of persistent inflammation are present, including an elevated Erythrocyte sedimentation rate (ESR) or CRP levels [2]. Compared with KD patients, KDSS patients had higher failure rate after first IVIG. Delayed treatment and more severe inflammation may make them more likely to be IVIG resistant [1,6]. Some studies showed that the combination of corticosteroids and IVIG may reduce the risk of coronary artery aneurysms in patients with severe form of KD [2,12].

## Conclusion

KDSS is a rare etiology for shock in childhood. The diagnosis could be missed because its atypical presentations. Cardiac prognosis depends essentially on early recognition of KDSS and initiation of adequate treatment.
